# Supplementary material for: Laboratory performance prediction using virtual reality behaviometrics
Source: PLoS One. 2022 Dec 19;17(12):e0279320. doi: 10.1371/journal.pone.0279320 (PMC9762586; doi:10.1371/journal.pone.0279320)
Supplement: S1 Table — Expertise metrics were used to calculate the participants’ expertise scores and divide them into novices and experts. (PDF) [file pone.0279320.s001.pdf]

S1 Table. Pre-test questionnaire. Expertise metrics were used to calculate the participants' expertise scores and divide them into novices and experts.

Pre-test expertise metric	Question
Occupation	**What is your occupation?** (Student at NEXT / Student at EUC / Operator at [biopharma company] / Equipment responsible at [biopharma company] / Other)
Training	**How often do you perform a pH calibration at your workplace?** (Every day / Multiple times per week / Once per week / A couple of times per month / Once per month / Less than once per month)<br><br>**When was the last time you received training on pH calibration at [biopharma company]?** (Less than half a year ago / Less than one year ago / Less than two years ago / More then two years ago / Never)<br><br>**What type of specific training on how to perform a pH calibration at [biopharma company] have you received in the past?** (I read the SOP, received one to one training, and took the eLearning before / I both read the SOP and took the eLearning before / I both read the SOP and received one to one training before / I only took the eLearning before / I only received one to one training before / I only read the SOP before / I never received training before)
Perceived prior knowledge	Please indicate how much you agree or disagree with the following statement: **I am familiar with pH calibration.** (I fully agree / I agree / I neither agree nor disagree / I disagree / I completely disagree)<br><br>Please indicate how much you agree or disagree with the following statement: **I know how to calibrate and adjust a pH meter**. (I fully agree / I agree / I neither agree nor disagree / I disagree / I completely disagree)<br><br>Please indicate how much you agree or disagree with the following statement: **I know how to document a pH calibration and adjustment according to the local SOP at [biopharma company].** (I fully agree / I agree / I neither agree nor disagree / I disagree / I completely disagree)
Prior knowledge	**Which important values do you have to transfer from the buffer bottle to the logbook during calibration?** (pH table value and expiration date / Error value and pH table value / Expiration date and error value / MPE and error value)<br><br>**You have just completed the first calibration before measurement. What do you have to do if the error value between the measured pH value and the table pH value on the buffer bottle is higher than MPE?** (Adjust the pH meter / Report a deviation / Contact the equipment responsible / Release the pH meter for use)<br><br>**After measurements, your calibration failed. What do you need to do now?** (Adjust the pH meter / Report a deviation / Contact the equipment responsible / Release the pH meter for use)

NEXT and EUC are production schools for vocational education in Denmark; SOP, standard operating procedure; MPE, maximum permissible error.
