# Supplementary material for: Laboratory performance prediction using virtual reality behaviometrics
Source: PLoS One. 2022 Dec 19;17(12):e0279320. doi: 10.1371/journal.pone.0279320 (PMC9762586; doi:10.1371/journal.pone.0279320)
Supplement: S2 Table — Model parameters were calculated for different sampling strategies. (PDF) [file pone.0279320.s002.pdf]

S2 Table. Overview of model parameters for the performance and reduced logistic regression models. Model parameters were calculated for different sampling strategies.

Model	Sampling strategy	Sensitivity	Specificity	Accuracy	AUC
Reduced logit model	None	0.61	0.88	0.77	0.79
	Random undersampling	0.74	0.79	0.77	0.79
	Random oversampling	0.74	0.79	0.77	0.80
Performance model	None	0.57	0.92	0.78	0.84
	Random undersampling	0.74	0.85	0.80	0.85
	Random oversampling	0.69	0.89	0.81	0.88

AUC, area under the curve.
