# Supplementary material for: Laboratory performance prediction using virtual reality behaviometrics
Source: PLoS One. 2022 Dec 19;17(12):e0279320. doi: 10.1371/journal.pone.0279320 (PMC9762586; doi:10.1371/journal.pone.0279320)
Supplement: S3 Table — No significant interactions were observed between age and expertise group. No significant main effects were observed for age. However, significant main effects were observed for the expertise group for both behaviometrics. (PDF) [file pone.0279320.s003.pdf]

S3 Table. Summary table of 2 x 2 ANCOVA comparing the influence of age and expertise group on the behavioral predictors used in the reduced logistic regression model. No significant interactions were observed between age and expertise group. No significant main effects were observed for age. However, significant main effects were observed for the expertise group for both behaviometrics.

**Behaviometric**	**Expertise group *P***	**Age *P***	**Interaction *P***
Challenge score	<0.001	0.34	0.33
Practical skill interactions	<0.001	0.42	0.15

ANCOVA, analysis of covariance.
