# Supplementary material for: Laboratory performance prediction using virtual reality behaviometrics
Source: PLoS One. 2022 Dec 19;17(12):e0279320. doi: 10.1371/journal.pone.0279320 (PMC9762586; doi:10.1371/journal.pone.0279320)
Supplement: Attachment — Submitted filename: MortenSommer_Response to Editor.pdf [file pone.0279320.s004.pdf]

June 14, 2022

Dear Dr. Heng Luo and Editorial Board of *PLOS ONE*,

Thank you for reviewing our manuscript entitled **"Laboratory performance prediction using virtual reality behaviometrics"**. We believe that the manuscript was marked as duplicate submission by the Editorial Manager system because it was previously included in Dr. Wismer's PhD thesis. Under Danish law, the thesis needs to be publicly available from the University's online library. Hence, we would like to highlight that the presented work has not been previously published in any academic journal. Given these circumstances, we would therefore appreciate if you were willing to re-evaluate our submission.

Sincerely,

Morten Sommer, PhD
Professor, Scientific Director
DTU Biosustain
Building 220, 2800 Kgs. Lyngby, Denmark
Email: msom@bio.dtu.dk
Phone: +45 21 51 83 40

Philip Wismer, PhD
Scientific Project Manager
DTU Biosustain
Building 220, 2800 Kgs. Lyngby, Denmark
Email: wismerp@alumni.ethz.ch
Phone: +45 50 14 53 99
