# Supplementary material for: Laboratory performance prediction using virtual reality behaviometrics
Source: PLoS One. 2022 Dec 19;17(12):e0279320. doi: 10.1371/journal.pone.0279320 (PMC9762586; doi:10.1371/journal.pone.0279320)
Supplement: Attachment — Submitted filename: Response to Reviewers_220907.docx [file pone.0279320.s005.docx]

# Response to Reviewer

## Reviewer #1

We thank the reviewer for their excellent comments, which we have addressed as detailed below to improve the manuscript:

**“In the “Procedure” section add an overview of the VR simulator of pH meter use.”**

We have added a short paragraph on the VR simulator that was used at the beginning of the “Procedure” section (lines 81-85).

“**Add some images for the tasks performed in VR simulation”**

We have included a new figure (Fig 1, line 100) showcasing the two tasks mentioned in the manuscript: flushing the pH electrode with water and evaluating different pH calibration points. We have added references to the figure on lines 89 and 93. The numbering of existing figures was adjusted accordingly.

**“Specify the participants’ age range and previous experience of using VR simulators”**

We have added the age range of participants on line 68: “pharmaceutical company employees (male: 37, female: 18; all age intervals above 20 years)” and line 71: “first-year students from two biopharma production schools (male: 20, female: 4; all age intervals from 10 to 50 years)”.

We have added the self-reported experience with VR as part of the metrics table (Table 1, line 119) and included the following reference on line 73: “78% of participants reported that they had never tried VR before participating in this study, while 22% had used occasionally.”

**“Indicate whether the VR simulator caused participants to experience any negative effects related to cybersickness.”**

The VR simulation was designed to reduce cybersickness. However, there were a couple of participants that could not continue the VR simulation for this reason. These participants were disregarded for the analysis. Line 108: “incomplete data records (e.g., if participants dropped out due to cybersickness) were excluded from the analysis.”

**“Provide some details about future work”**

We added a new paragraph at the end of the discussion section to address limitations and further directions (lines 262-271).
